# Tactile stimulation reduces aggressiveness but does not lower stress in a territorial fish

**DOI:** 10.1038/s41598-018-36876-1

**Published:** 2019-01-10

**Authors:** Marcela Cesar Bolognesi, Ana Carolina dos Santos Gauy, Eliane Gonçalves-de-Freitas

**Affiliations:** 1 Departamento de Zoologia e Botânica, Instituto de Biociências, Letras e Ciências Exatas, Universidade Estadual Paulista (UNESP), Cristóvão Colombo, 2265, 15054-000 São José do Rio Preto, SP Brazil; 2 Centro de Aquicultura da UNESP, São José do Rio Preto, SP Brazil

## Abstract

Body tactile stimulation has a positive effect upon highly social animals, such as mammals and cleaner-client coral-reef fish, by relieving stress and improving health. Conversely, some tactile contacts are naturally detrimental, such as those resulted from aggressive interactions. To study whether positive responses from tactile stimulation are generalized among vertebrates, we tested its effect on stress response and aggressive behavior in a territorial fish species, Nile tilapia. We developed an apparatus made of a row of sticks bordered by silicone bristles that was positioned in the middle of the aquarium, and through which fish had to pass to access food, thus receiving tactile stimulation. Isolated fish experienced tactile stimulation for 7 days, and  were assigned to 2 types of stressors: non-social (confinement) or social (aggressive interaction). Each of them had a corresponding control treatment without tactile stimulation. Although fish spontaneously crossed the apparatus, we did not observe a decrease in plasma cortisol levels immediately after stressor application as a response to the use of the apparatus, either for social or non-social treatment. However, tactile stimulation reduced aggressive interaction in the social treatment, showing a positive effect on a territorial fish species, and pointing to a way to improve welfare.

## Introduction

Tactile stimulation (like scratches and touches) is the mechanical contact between two or more individuals of the same or different animal species that are perceived either as a positive or a negative interaction. The positive effect of tactile stimulation on mammals, including humans, is known when physical contact is performed as massage therapy. In this interaction, the tactile stimulation relieves stress^1^ and increases serotonin levels^1^, thus having positive effects on health. Piglets^2^, lambs^3^ and dairy cows^4^ experience lower heart rates, and cattle^5^ show lower cortisol levels when scratched and stroked by humans. Such positive effects can also be artificially achieved. In a cleaner-client fish interaction, for instance, Soares *et al*.^6^ observed that the tactile stimulation performed by a coral-reef cleaner fish reduces the stress level in the client fish *Ctenochaetus striatus*. The authors developed an apparatus composed of a cleaner fish model that mechanically rubbed the client fish, thus confirming that positive effects can be achieved even by inanimate objects.

Most positive responses to tactile stimulation are associated with animals’ natural behavioral repertoire. Physical contact between individuals, such as allo-grooming in primates^7^ and cleaner-client interactions among coral-reef fish^8^, are part of the natural behavior of these animals. Both types of interaction are adaptive, respectively, to keep social groups together by bonding individuals^7^, and to keep the cooperation in the cleaner-client fish mutualism. Conversely, several types of tactile contact are naturally detrimental, such as agonistic physical interactions, which may cause injuries, pain and social stress^9^ to individuals. Therefore, the tactile stimulation could be a negative stimulus for species whose natural behavior is characterized by territorial defense and social hierarchy, such as cichlid fish.

Many cichlid species are commonly found in fish farming activities, being frequently subjected to stressful situations, such as net catching^10^, overpopulation^11^, confinement^12^, and grading^13^. Furthermore, there are other conditions which may lead to aggressive interactions among them increasing the stress levels and detrimental effects on their health^14^, including increased probability of mortality^15^.

**Figure 1:**
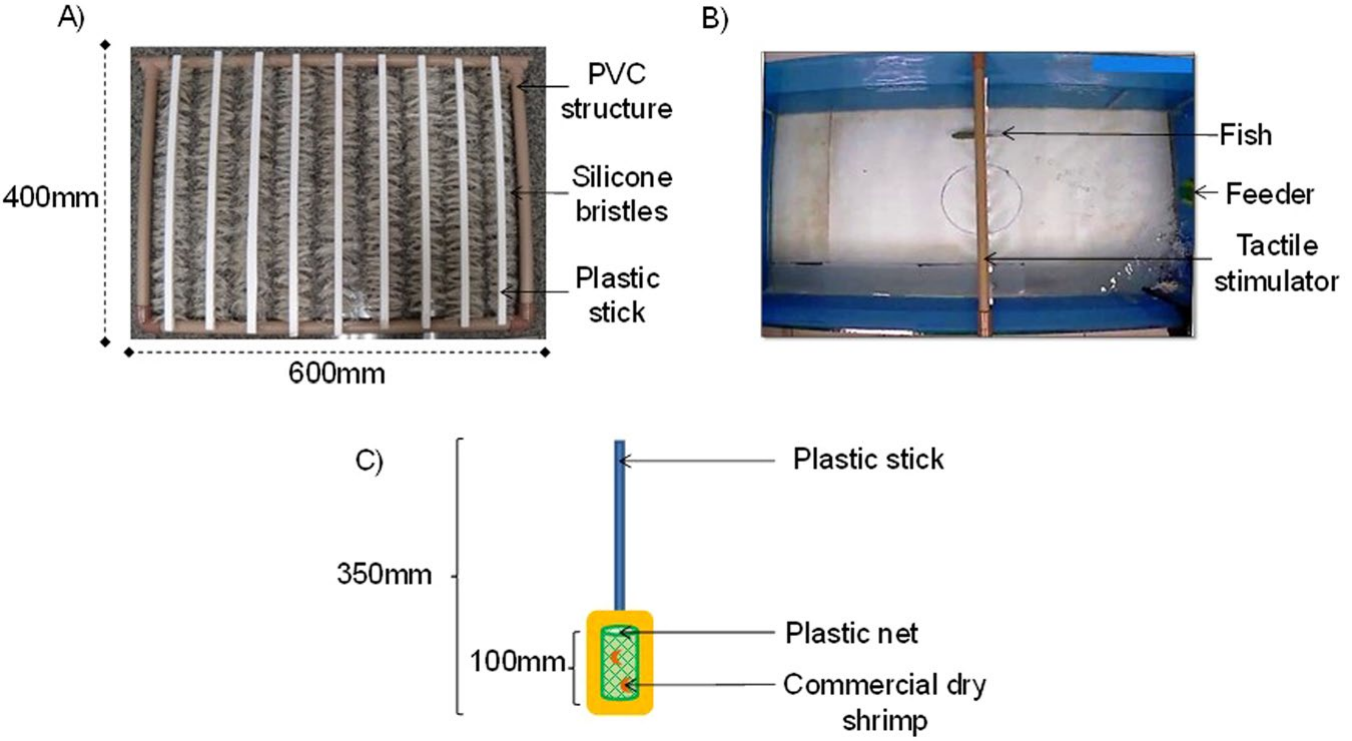
Photo of the apparatus developed for tactile stimulation, showing the PVC structure and silicone bristles (**A**) through which fish passed to reach food, receiving tactile stimulation as a consequence (from above in **B**). Feeder is shown in detail (**C**), with dry shrimp trapped in the small plastic net.

During stressful situations, the higher activity of the hypothalamic-pituitary-adrenal axis (interrenal cells in fishes) leads to an increased circulating glucocorticoid levels (as cortisol)^16^. Although stress is considered an adaptive response to environmental challenges^17^, if the stressor is intense or lingering, animals may undergo a chronic stress state (distress), characterized by high cortisol level and marked by depression of the immune system^18^, decrease in growth rates^19^, and impairment of reproduction^14^. Distress also causes neuron death^20^ with consequent reductions in the cognitive ability. All those effects are undesirable, both for fish culture and for fish welfare. As most handling methods for husbandry fish in rearing environments are still unavoidable, finding ways to relieve any kind of distress is important to promote animal welfare. Anecdotal information about fish pets indicates that, even for cichlids, tactile stimulation could be positive, since fish seem to choose being touched or petted by human owners (see ref.^21^). Empiric data, however, is unavailable. In fact, some degree of positive tactile stimulation probably occurs during cichlids courtship behavior, when male and female touch each other’s body several times by a less intense biting, quivering and lateral undulations^22^. In this moment, tactile stimulation cannot be negative, otherwise male and female would avoid each other and mating would be impaired, being disadvantageous to Darwinian fitness. In this study, we tested whether tactile stimulation has a positive effect on the cichlid fish Nile tilapia, *Oreochromis niloticus* (L.), a territorial species highly farmed in the world, and a widespread model to study mechanisms underlying fish behavior. We tested the effect of tactile stimulation on Nile tilapia that underwent confinement (non-social stressor), often applied in fish farming^12^, and aggressive interactions (social stressor), which is part of the cichid’s natural behavior, but can be exacerbated under some rearing conditions^23^. We developed an apparatus consisting in a row of sticks with silicone bristles by their sides (Fig. 1A) placed in the middle of the aquarium (Fig. 1B). Fish had to pass through it to access food, which was provided by a feeder (Fig. 1C), thus receiving tactile stimulation. We predicted that, if tactile stimulation alleviates stress, fish would search for more stimulus after being exposed to stressors. In addition, tactile stimulation would relieve stress by reducing cortisol levels.

There are only two other studies conerning the positive effects of tactile stimulation on fish^6,24^, to the best of our knowledge, a very modest number when compared to the knowledge about mammals. Thus, studies about the functions and effects involved with tactile stimulation in fish can help us understand the evolution of this mechanism in vertebrates.

## Results

### The apparatus worked

**Figure 2:**
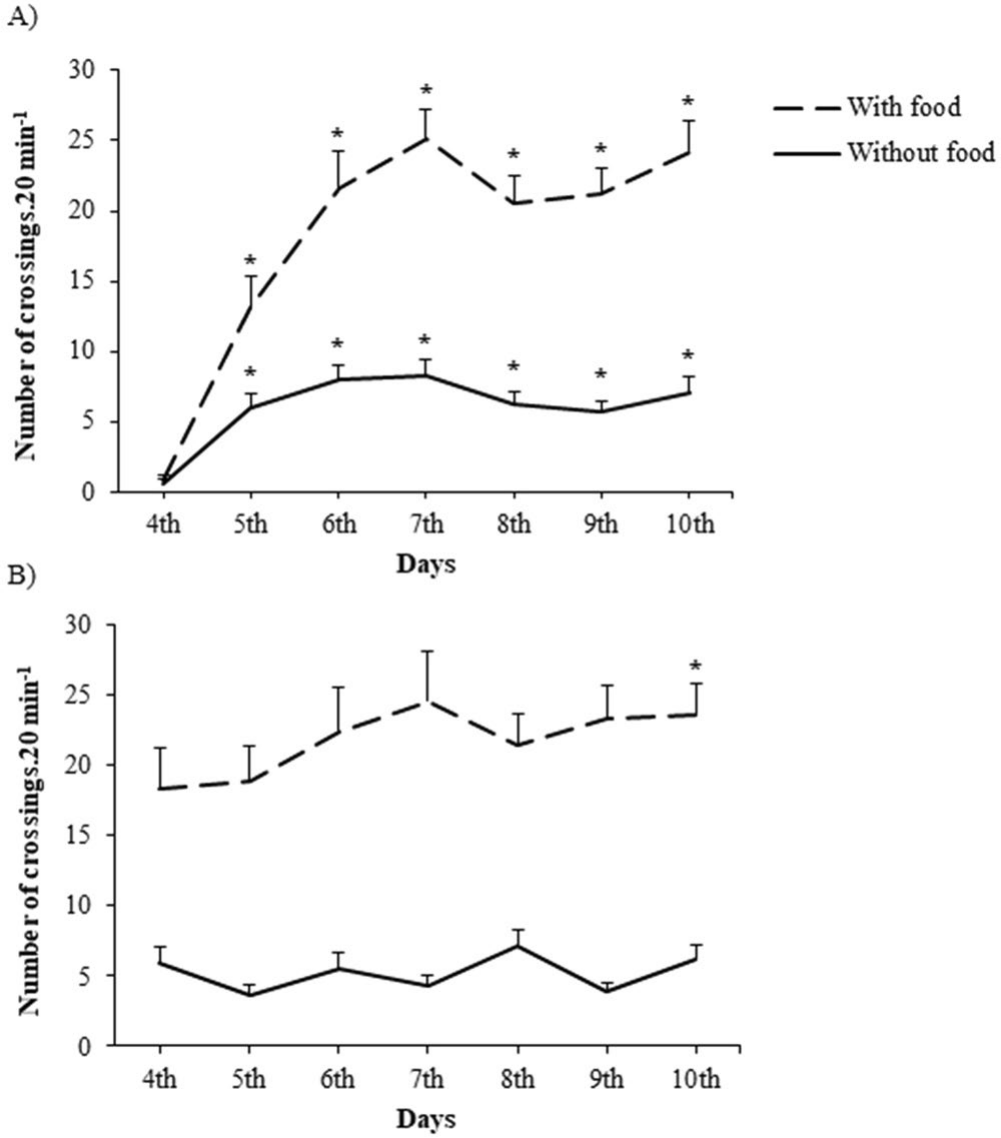
Number of crossings through the center of the aquarium during period with/without food in the treatment with tactile stimulation in both studies, then 15 replica for Non-social stress experiment and 14 replica for Social stress experiment were observed together (**A**, N = 29) and in treatment without tactile stimulation (**B**, N = 29). Asterisk indicates a significant difference from apparatus introduction, that is, 4th day (p < 0.003) after Mixed model ANOVA, followed by Tuckey-HSD post hoc test. Data are mean ± SE.

We assumed that the apparatus would fit our goal whether fish passed through it spontaneously. We compared the number of crossings through the apparatus (20 min/day) both in the presence and absence of food, considering spontaneity the act of crossing the apparatus in the absence of food. Fish went through the apparatus under both conditions, although less frequently in the absence than in the presence of food (interaction between presence/absence of food and days: F_(6,336)_ = 6.24, p = 0.00003; Fig. 2A). In the treatment without tactile stimulation, fish crossed the center of the aquarium less frequently in the absence of food (presence *vs* absence of food: F_(1,56)_ = 41.58, p < 0.0001; among days: F_(6,336)_ = 3.85, p = 0.001; no statistic interaction: F_(6,336)_ = 1.37, p = 0.22; Fig. 2B). We looked for a soft material to avoid body lesions and injuries due to physical contact with the stimulation, therefore we chose silicone bristles. Nevertheless, in the end of experiments, each animal was carefully examined to check for possible body lesions or any kind of injury caused by the tactile stimulation. We did not observe any scale loss or body injuries from fish contact with the silicone bristles.

### Fish went through the apparatus after stress

**Figure 3:**
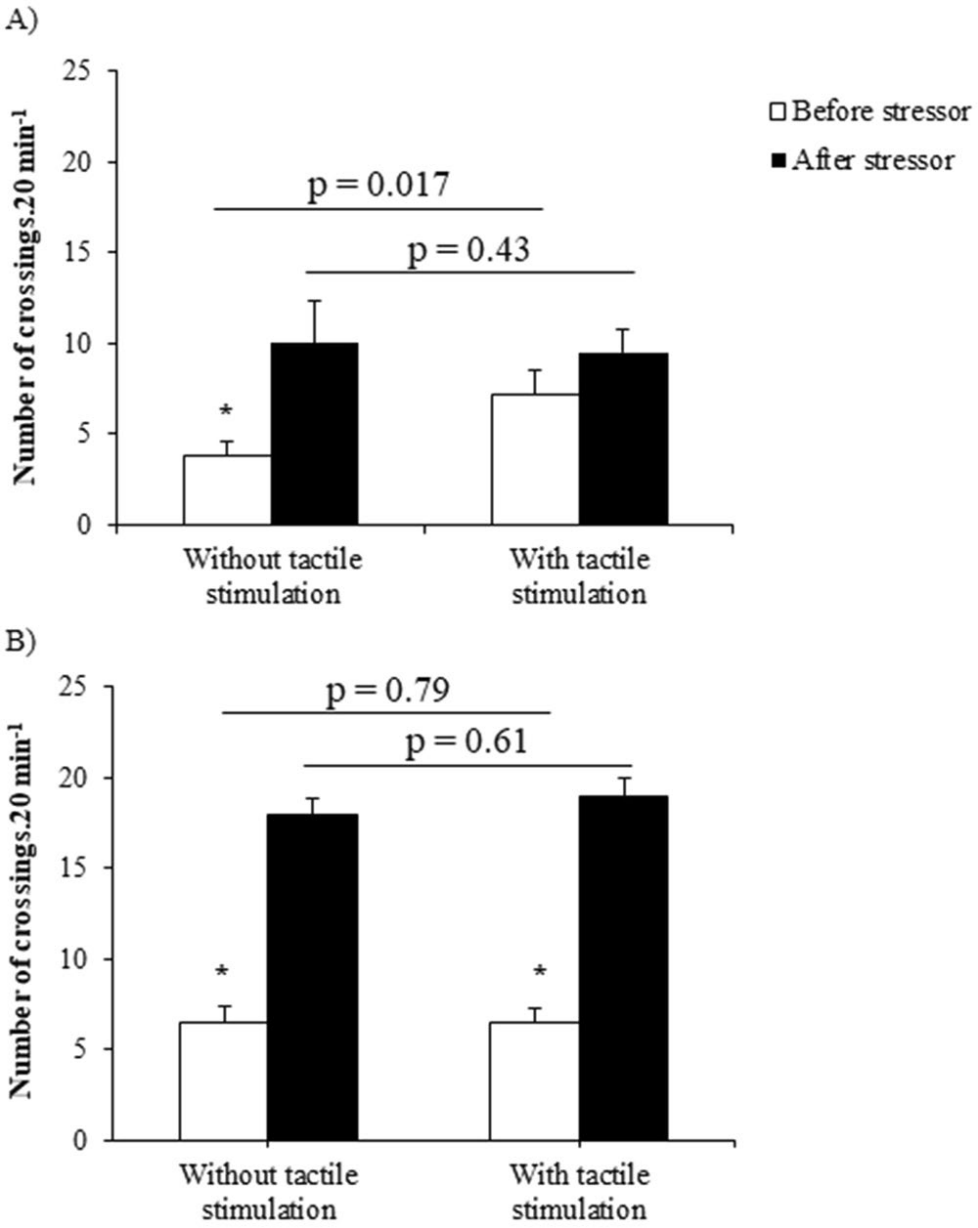
Number of crossings before and after (**A**) non-social (N = 15) and (**B**) social stressors (N = 14) in both treatments. P values on upper line compare between treatments, and asterisk indicates significant differences within treatments after planned comparisons. Data are shown as mean ± SE.

To test whether fish would use the apparatus as a way to alleviate stress, we compared the number of crossings through it right after the first stressing session with the mean number of crossings from the 7 previous days. ANOVA main results showed no significant interaction (between and within treatments: F_(1,28)_ = 1.29, p = 0.26; Fig. 3A). However, a marginally significant difference was found between treatments (F_(1,28)_ = 4.11, p = 0.052), and a significant one within treatments (F_(1,28)_ = 7.73, p = 0.01). Planned comparisons showed that the number of crossings was different before stressor between treatments (F_(1,28)_ = 6.36, p = 0.017), and it was similar after stressor (F_(1,28)_ = 0.63, p = 0.43). Fish crossed the center of the aquarium more frequently after the stressor in the treatment without tactile stimulation (F_(1,28)_ = 7.68, p = 0.009), and with similar frequency in the treatment with tactile stimulation (F_(1,28)_ = 1.34, p = 0.25).

After aggressive interactions, there was no significant difference in ANOVA interaction between and within treatments (F_(1,26)_ = 0.04, p = 0.84; Fig. 3B) and neither between treatments (F_(1,26)_ = 0.22, p = 0.64); but we found difference within treatments (F_(1,26)_ = 66.41, p < 0.0001). Planned comparisons showed similar number of crossings between treatments before (F_(1,26)_ = 0.06, p = 0.79) and after stressor (F_(1,26)_ = 0.26, p = 0.61). Fish went 4 times more often through the apparatus or crossed the center of the aquarium than before stressor in treatment with tactile stimulation (F_(1,26)_ = 34.91, p < 0.0001) and treatment without tactile stimulation (F_(1,26)_ = 31.53, p < 0.0001).

### Aggressive interaction was lower in treatment with tactile stimulation

**Figure 4:**
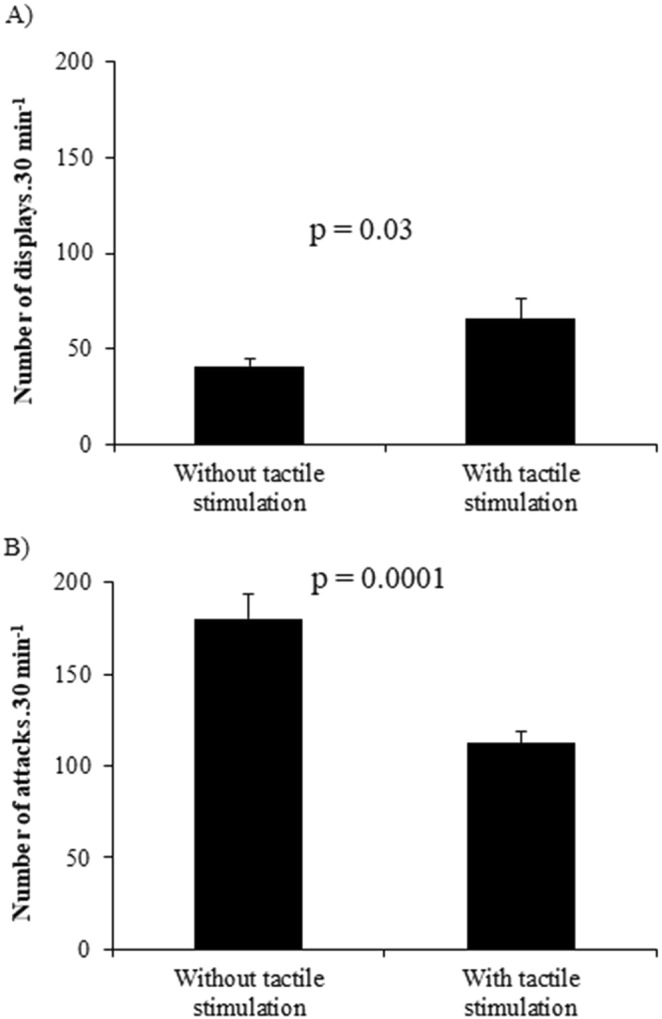
Number of displays (**A**) and attacks (**B**) in treatment with tactile stimulation (N = 14) and treatment without tactile stimulation (N = 14). P values on upper line show differences between treatments after unpaired t-test. Data are shown as mean ± SE.

Aggressive interaction was affected by tactile stimulation. Animals in the treatment with tactile stimulation showed more displays (t = −2.22, p = 0.03; Fig. 4A) and less attacks (t = 4.45, p = 0.0001; Fig. 4B) than animals without tactile stimulation. There was no correlation between displays or attacks with crossings through the apparatus (r = −0.002, p = 0.99 and r = 0.16 and p = 0.6, respectively).

### Cortisol was not lower in treatment with tactile stimulation

**Figure 5:**
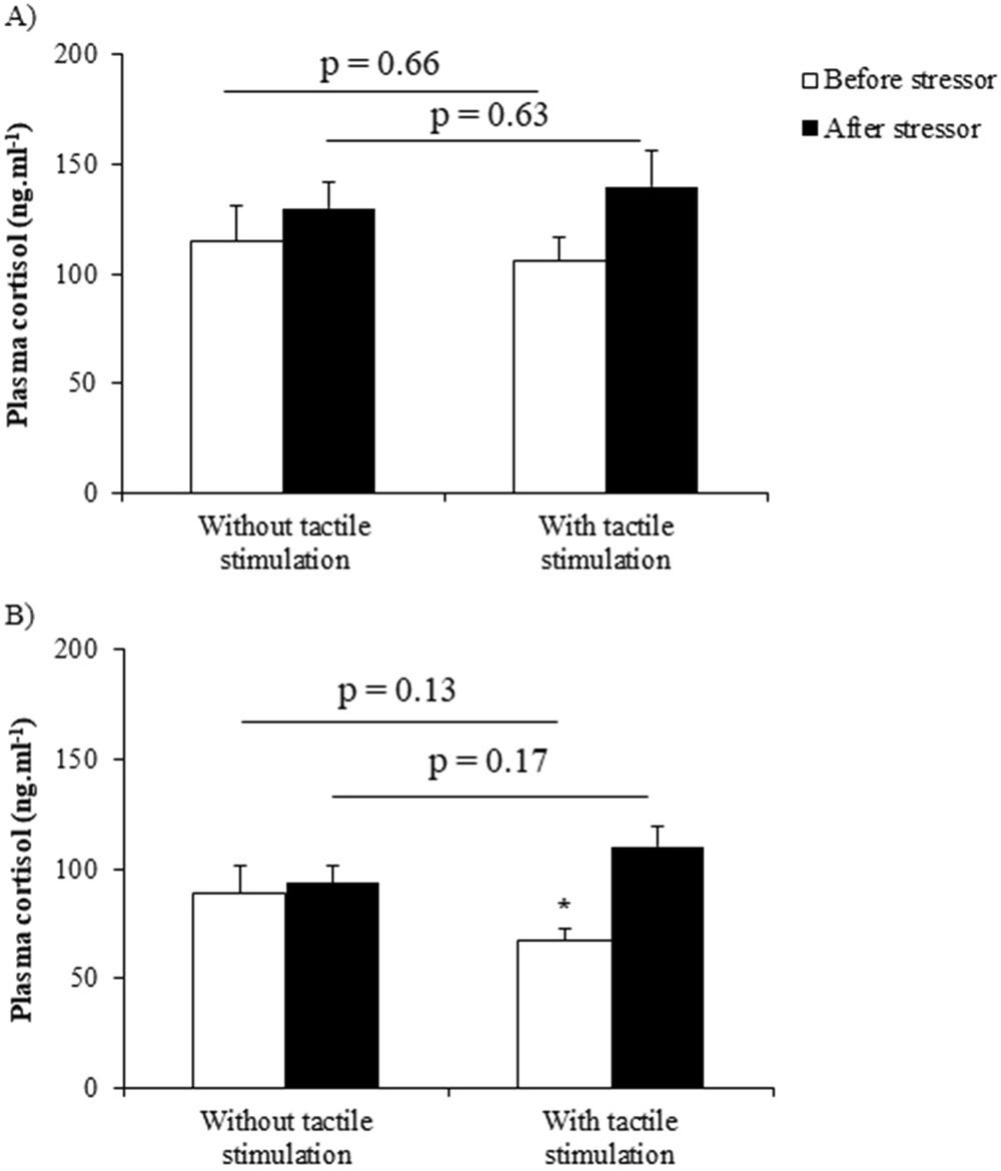
Plasma cortisol level before and after (**A**) non-social (N = 10) and (**B**) social stressor (N = 9). P values on upper line compare between treatments, and asterisk indicates significant differences within treatments after planned comparisons. Data are shown as mean ± SE.

Plasma cortisol level was not reduced by tactile stimulation in the confinement experiment. It was similar between treatments with and without tactile stimulation, as well as within sampling days (ANOVA interaction between and within treatments: F_(1,18)_ = 0.39, p = 0.53; within treatments: F_(1,18)_ = 2.73, p = 0.11; between treatments: F_(1,18)_ = 0.39, p = 0.53; Fig. 5A). Planned comparisons showed that cortisol level was similar before (F_(1,18)_ = 0.19, p = 0.66) and after stressor (F_(1,18)_ = 0.23, p = 0.63) between treatments. Cortisol levels were also similar before and after stressor in treatments with (F_(1,18)_ = 2.61, p = 0.12) and without (F_(1,18)_ = 0.52, p = 0.47) tactile stimulation. Furthermore, a significant difference was found for cortisol levels after aggressive contests (interaction between and within treatment: F_(1,16)_ = 5.77, p = 0.03; Fig. 5B), although there were no differences between treatments (F_(1,16)_ = 0.05, p = 0.82). Planned comparisons showed that cortisol level was similar before (F_(1,16)_ = 2.49, p = 0.13) and after stressor (F_(1,16)_ = 2.05, p = 0.17) between treatments. Cortisol level was lower before stressor than after stressor in treatment with tactile stimulation (F_(1,16)_ = 14.62, p = 0.001) and was similar in treatment without tactile stimulation (F_(1,16)_ = 0.18, p = 0.67).

There were no significant correlations either between the number of crossings after confinement and cortisol levels (r = −0.35, p = 0.32), or between the mean of crossings on the 7 days before stress and cortisol levels (r = −0.3015; p = 0.4). We did not find a correlation between crossings and cortisol levels after aggressive interactions as well (r = 0.17, p = 0.65; r = 0.13, p = 0.72, crossings before and after stressor, respectively).

We did not find a significant correlation between the number of displays and cortisol levels after social stress, either for the treatment with tactile stimulation (r = 0.31, p = 0.41) or for the treatment without tactile stimulation (r = 0.07, p = 0.84). However, there was a positive correlation between the number of attacks and cortisol after stress following tactile stimulation (r = 0.69, p = 0.04), whereas a non-significant correlation was observed in the treatment without tactile stimulation (r = 0.04, p = 0.91).

## Discussion

In this study we successfully created a protocol for providing fish with tactile stimulation. Such stimulation did not change cortisol levels on Nile tilapia immediately after stressor application, but it was effective in decreasing aggressiveness, thus probably bringing about positive effects on fish’s welfare.

We considered the apparatus efficient in providing fish with body tactile stimulation because they spontaneously went through it after training. In both treatments, (with and without tactile stimulation) food stimulated fish locomotion, but the frequency of crossings before and after feeding indicates that fish use the stimulator apparatus irrespective of food presence. In the treatment without tactile stimulation, fish were able to cross the center of the aquarium since the first day of observation, whereas in treatment with tactile stimulation they hardly crossed through the apparatus on the first day; the number of crossings increased gradually up to the third day of experiment. This shows that some learning processes was required to deal with the new structure inside the aquarium since the apparatus was initially avoided but, after that, the stimulator did not affect the fish’s freedom in swimming and exploring their environment. In addition, tactile stimulation seemed to be soft enough to avoid body injuries or scale loss, which would be a gateway for pathogens. Therefore, our protocol is suitable to test several hypotheses regarding tactile stimulation in fish.

After a stressful experience, animals tend to escape from the detrimental stimulus. It is supposed that animals can also search for ways to alleviate stress (e.g., coping with stress by performing stereotyped behaviors^25^) and show strong motivation to access preferred items in the environment, while avoiding non-preferred ones^26^. Therefore, we expected there would be an increase in the number of crossing through the apparatus after fish were subjected to stressors, thus indicating that body tactile stimulation could alleviate stress in fish. Even though it was not observed after confinement, it was evident after social stress and, at first, it could be interpreted as a motivational behavior for receiving tactile stimulation. However, there was also an increase in crossing the center of the aquarium in the treatment without tactile stimulation, at the same intensity; thus, crossing through the apparatus was a consequence of fish’s increased locomotion after fighting. This behavior did not seem to be a stereotyped activity because the high rate of locomotion was not rhythmically repetitive^25^. Nevertheless, it seemed to be a type of displacement behavior observed in fish after aggressive interactions^27^. Since in our study there were no elements in the environment to redirect aggression^27^, motivation to fight could be turned into swimming activity.

Greater amount of physical activity increases the levels of endorphin^28^ and of other opioid substances^29^, which are neurotransmitters associated with reducing anxiety and stress^30^, and could be effective in decreasing aggressive behavior. This mechanism, however, does not explain our data (reduced aggressive interaction), since fish’s locomotion was similar in both the treatments with and without tactile stimulation. However, it is known that body tactile stimulation increases serotonin levels in humans and other mammals^1^, and serotonin does present an inhibitory effect on the aggressive behavior in fish^31,32^ and other vertebrates. Then, we infer that tactile stimulation may reduce aggressiveness by increasing serotonin levels, although this mechanism should yet be tested.

An animal’s response to a determined stimulus is based on a capacity evolutionary selected for such response. Thereby, the natural response to some positive effect from tactile stimulation probably was selected in social fish species, particularly for courtship behavior^22^. In this moment, aggressiveness shoud be reduced to allow mating.

Despite the association found between tactile stimulation and aggressive interaction, stress was not reduced, which suggested some possible non-exclusive explanations: 1. tactile stimulation does not reduce stress in this territorial fish species because positive body tactile stimulation is not associated to its natural repertoire. For instance, stress is reduced in *Ctenochaetus striatus*^6^, a fish species that naturally receives frequent tactile stimulation from the cleaner fish *Labroides dimidiatus*^8^. 2. The effect exists, but longer stimulation would be required. 3. Cortisol levels were already very high, due to social isolation. In fact, the animals presented high levels of cortisol in contrast to basal levels reported to GIFT Nile tilapia^23^, which could be explained by social isolation^33,34^. Fish could be already stressed therefore it precluded the observation of significant increment on plasma cortisol after non-social stress. Although a higher cortisol level was shown after social stress, there was no difference from the treatment without tactile stimulation, thus suggesting there was an effect of social isolation on the fish response which was not counteracted by tactile stimulation. 4. Blood sampling occurred during cortisol peak (see ref.^35^). As we designed a study to be compared to that by Soares *et al*.^6^, we sampled blood right after the stressor application. Thus, we had no data regarding the possible effect of tactile stimulation on cortisol levels after peak elapsed time. In this sense, it is important to evaluate the effect of tactile stimulation during the stress-recovering stage from social interaction (see ref.^36^).

Although cortisol level is the main stress indicator, it can also be involved in other functions, for instance, in inflammatory/immune responses to mechanical damages on tissues, and other functions that requires a metabolic changing^16^. Moreover, cortisol is not always the best physiological marker for stress in highly social species^37^. Then, a higher set of physiological indicators, as well gene expression in specific brain areas could be necessary to evaluate the stress in a broader sense^38^. Thus, we have to take in account that the cortisol solely brought a limitation for understanding the entire scenario related to Nile tilapia’s stress response to tactile stimulation. An associated pattern with a positive correlation between the number of attacks and cortisol levels was found after tactile stimulation, which was non-significant in the treatment without tactile stimulation (dissociated pattern). Some differences in the social environment can cause differences between associated/dissociated hormones and behavior patterns in cichlids^39^. Therefore, tactile stimulation possibly affects the HPI axis, associating it with social stress.

Studies carried out with domestic mammals are usually combined with human presence, and stimulation is provided by touch^5,40^, petting or stroking^2–4,40^. Such interactions show both positive and negative effects on the animals. Certain studies focus on the affection and bonds that are created between humans and animals^41–43^, so they do not eliminate the social context of human contact, which may influence the animal’s perception. In this study, tactile stimulation was isolated from the human presence, therefore highlighting the potential of tactile stimulation alone for reducing aggressiveness in Nile tilapia, which may lead to improved welfare.

## Methods

### Fish housing

Adult male Nile tilapia specimens from the Aquaculture Center of UNESP (CAUNESP) in Jaboticabal, SP, Brazil, were kept in outdoor ponds at the Laboratory of Animal Behavior, UNESP, São José do Rio Preto. They were selected for the study and taken to the laboratory where they were acclimated for 20 days in polyethylene water tanks (ca. 500 L, 1 fish/10 L) at 27 °C and light regime from 7:00 a.m. to 7:00 p.m. Fish were fed with food for tropical fish (28% CP, apparent satiety) twice a day (9:00 a.m. and 3:00 p.m.). Water quality was maintained using biological filters (400 L/h) and constant aeration.

### Experimental Design

Nile tilapia males were individually assigned to one aquarium equipped with an apparatus developed to provide them with body tactile stimulation (Fig. 1A). Fish were tested for one out of two stressors: confinement (non-social stressor) and aggressive interaction (social stressor). For each treatment, we had a control one without tactile stimulation.

### Tactile stimulation

We developed an apparatus made of a rectangular polyvinyl chloride (PVC) pipe structure, filled with vertical sticks bordered by silicone bristles (Fig. 1A). We chose silicone bristles because of their softness, so they would not remove mucus from the fish’s skin or injure their bodies. The apparatus was inserted in the center of the aquarium so that fish had to pass through the bristled sticks to access food (Fig. 1B), which in turn was placed on the opposite side of the aquarium by a handle feeder. The feeder was a plastic stick with a plastic net attached in its end, wherein food was trapped (Fig. 1C).

### Protocol

Isolated fish individuals were adjusted in the aquaria for 3 days before introducing the apparatus. During those days, fish were fed with dry shrimp at 9:00 a.m. and 3:00 p.m., by using the feeder. The same was done in the treatment without tactile stimulation. Animals that did not eat during this period were withdrawn from the study.

After the adjustments, the apparatus was introduced in the center of the aquarium and remained there for 7 days. Fish were video-recorded daily to quantify the number of crossings through the apparatus; 5 min before, 10 min during and 5 min after the introduction of the feeder. Records started at 8:00 a.m. and at 2:00 p.m. On the 11^th^ day, fish were subjected to stressors twice, the first record being made to quantify the number of crossings after stress, and the second one to quantify cortisol right after stress, so that it was possible to compare data with those by Soares *et al*.^6^. Blood samples were obtained on the 3^rd^ day for evaluating the baseline plasma cortisol levels and on the 11^th^ day for checking the effect of tactile stimulation on stress response. The same procedure was repeated in the treatments without tactile stimulation. This protocol was the baseline for testing both social (aggressive interaction) and non-social (confinement) stress, which were run independently. The protocol timeline is summarized in Table 1.

**Table 1:** Protocol timeline.

Days → Time ↓	Adjustment to the aquarium			Tactile stimulation Period							Stress test
	1^st^	2^nd^	3^rd^	4^th^	5^th^	6^th^	7^th^	8^th^	9^th^	10^th^	11^th^
8:00 AM	Fish isolation			Introduction of the apparatus VR1	VR 3	VR 5	VR 7	VR 9	VR 11	VR 13	– 1^st^ stress test (30 min) – Video recording of fish crossing through the apparatus (20 min)
02:00 PM			1^st^ blood sample	VR 2	VR 4	VR 6	VR 8	VR 10	VR 12	VR 14	– 2^nd^ stress test (30 min) – 2^nd^ blood sample

The sequence of events during the 11-day experimental protocol started with fish isolation to adjustment to aquaria (3 days). The following 7 days were assigned to allow contact of fish with the apparatus for tactile stimulation. In the end of the period (11^th^ day), fish were addressed to either a social (aggressive interaction in pairs) or non-social (confinement) stress test. Tests were run independently for social (N = 15) and non-social (N = 14) stress, each one having a control treatment without tactile stimulation. VR means video-recording.

### Non-social stress experiment

In the morning of the 11^th^ day, fish were subjected to the confinement stress test, which consisted of reducing in 90% the aquarium space using an opaque plate, thus confining the animal to one end of the aquarium for 30 min (e.g., ref.^44^). This stressor has already been tested for Nile tilapia^44,45^. After the confinement, the plate was removed, and fish were video-recorded (20 min) to quantify the number of crossings through the apparatus. As the crossings could be affected by fish locomotion in the aquarium, we recorded the number of times fish crossed through the center of the aquarium in the treatment without tactile stimulation. The stressor was applied again after 6 hours (in the afternoon), followed by blood sampling for plasma cortisol assay. In the treatment without tactile stimulation, the animals underwent the same procedure described, yet without the presence of the apparatus in the aquaria. Fifteen isolated males were tested in each treatment both with and without tactile stimulation.

### Social stress experiment

In the morning of the 11^th^ day, fish were subjected to the aggressive interaction test. Two isolated individuals were removed from their aquaria and were paired in a new aquarium (40 × 30 × 40 cm ca. 48 L) to avoid prior residence effect^46^. Two fish of the same treatment and with similar size were paired as they underwent the same procedures (absence or presence of tactile stimulation). Aggressive interaction was video-recorded for 30 min, then, each fish was returned to its original aquarium and was video-recorded (20 min) to quantity the number of crossings through the apparatus. As in the non-social experiment, we also recorded the number of times fish crossed through the center of the aquarium in the treatment without tactile stimulation. Fish were paired again in the afternoon for aggressive interaction video-recording (30 min), and a blood sampling for plasma cortisol assay was performed. Different individuals (from the first encounter) were paired to avoid the effect of the previous experience^47^. In the treatment without tactile stimulation the animals underwent the same procedure, yet without the insertion of the apparatus. Fourteen isolated males were tested in each treatment, both with and without tactile stimulation.

### Behavioral data

In the social stress experiment, fish were individually identified by a red visible implanted elastomer (VIE tags), inserted under 2 or 3 scales on each side of the body. Aggressive interactions were quantified according to refs^48–50^. Aggressive behavior was labeled as attacks or displays. Attacks are the interactions with physical contact and associated with high energy expenditure (chase, nipping, lateral fight, undulation and mouth fight); on the other hand, displays are aggressive interactions without physical contact and associated with less energy expenditure^51^ (lateral threat, perpendicular threat and circling).

### Cortisol assay

In fish stress is marked by an activation of two main axes. The first is the hypothalamus-sympathetic-chromaffin cells axis, which release the neurohormones norepinephrine and epinephrine, whose trigger a fast response to increased energy demand^52^. The second is the hypothalamic–pituitary–interrenal tissue (HPI) axis^52^. In HPI axis the corticotrophin releasing hormone (CRH) released from the hypothalamus stimulates the anterior pituitary gland to release adrenocorticotrophic hormone (ACTH) that, in its turn, stimulate the interrenal tissue to increase the glucocorticoid hormones releasing, being cortisol the main corticosteroid in teleost fishes^52^. This increment in cortisol levels in response to a stressor is a primary indicator of stress levels in fishes^16^, although some caution should be considered when using only one marker.

After being anesthetized by immersion in Benzocaine (0.09 g.L^−1^), a fish blood sample was collected from the caudal vein by hypodermic needles and heparinized syringes. The blood sampling of each fish lasted less than 2 minutes to avoid interference of manipulation in the results^18^. Blood was centrifuged at 3,000 rpm for 10 min and the plasma was frozen at −20 °C for further cortisol assays. Cortisol was evaluated through ELISA - Enzyme Linked Immunosorbent Assay, using commercial kits (IBL - Immuno Biological Laboratories, Hamburg, Germany). The intra-assay CV = 3.32%, 6.51% and 4.62% in each plate, and the inter-assay CV = 6.81%.

### Experimental details

Before isolation, animals were anesthetized by immersion in Benzocaine (0.03 g.L^−1^), weighed, measured and tagged with an elastomer. Fish mean (±S.E.) standard length and weight were respectively: Non-social experiment – Treatment with tactile stimulation: 11.44 cm ± 0.92 cm; 47.26 g ± 11.15 g (N = 15); Treatment without tactile stimulation: 11.74 cm ± 0.90 cm; 51.33 g ± 10.62 g (N = 15). Social stress experiment: – Treatment with tactile stimulation: 11.42 cm ± 0.52 cm; 51.83 g ± 8.76 g (N = 14); Treatment without tactile stimulation: 10.97 cm ± 0.41 cm; 48.20 g ± 6.55 g (N = 14). We did not find significant differences in the biometric data between treatments (Non-social experiment: independent t-test, t = −0.70, p = 0.49, standard length; t = −0.73, p = 0.47, weight. Social stress experiment: t = 1.85, p = 0.07, standard length; t = 1.21, p = 0.23, weight). At the end of the experiment all fish were killed (Benzocaine 0.18 g.L^−1^) and opened for gonadal inspection. Adult male Nile tilapia present developed testicles filled with semen, thus showing an opaque white color^53^. We confirmed macroscopically that all individuals were adult males.

Animals were observed in glass aquaria (120 × 60 × 40 cm, containing 140 L of water, since this quantity was enough for a single fish) coated with blue plastic to avoid visual contact with animals in neighboring aquaria. Video-recording was made with cameras placed above the aquaria, which sent the records to a computer in an adjacent room. The photoperiod was set to 12 L:12D (from 7:00 a.m. to 7:00 p.m.) and temperature to 27 °C. Water quality was monitored by commercial kits and electronic devices and given by mean ± SE: Ammonia (0.025 ± 0.019 ppm); Nitrite (0.125 ± 0.072 ppm); pH (7.36 ± 0.11).

### Statistical analysis

Data were tested for outliers by Grubbs test and those found were replaced by the mean^54^. The data were, then, checked for normality by Kolmogorov-Smirnov test and homoscedasticity by Fmax^55^. When necessary, data were transformed by log (x + 1) to fit parametric assumptions. In both social and non-social experiments, a mixed-model ANOVA was used to compare between (with tactile stimulations *vs* without tactile stimulation) and repeated data for the following variables: number of crossings/min through the center of the aquarium in periods with and without food); number of crossings/min before and after stress test in both treatments; plasma cortisol concentrations on the 3^rd^ and 11^th^ days. Tukey-HSD post hoc test was applied when necessary. Planned comparisons were used to contrast between and within treatments. T-test was used to compare the number of attacks and displays between treatments. Correlations between the number of crossings and the cortisol levels were checked by Pearson’s correlation test. Statistical significance was set at p ≤ 0.05.

### Ethical note

This study followed the Animal Behavior Society guidelines for animal usage in research (2012) and was in accordance with the Ethical Principles on Animal Experimentation adopted by the National Council for the Control of Animal Experimentation (CONCEA/Brazil). It was approved by the Committee on Ethics in Animal Use, IBILCE, UNESP, São José do Rio Preto, permit #129/2016.
